# *Prototheca zopfii* Induced Ultrastructural Features Associated with Apoptosis in Bovine Mammary Epithelial Cells

**DOI:** 10.3389/fcimb.2017.00299

**Published:** 2017-07-13

**Authors:** Muhammad Shahid, Jianfang Wang, Xiaolong Gu, Wei Chen, Tariq Ali, Jian Gao, Dandan Han, Rui Yang, Séamus Fanning, Bo Han

**Affiliations:** ^1^College of Veterinary Medicine, China Agricultural University Beijing, China; ^2^Beijing Key Laboratory of Traditional Chinese Veterinary Medicine, Beijing University of Agriculture Beijing, China; ^3^Beijing Key Laboratory for Agricultural Application and New Technique, Beijing University of Agriculture Beijing, China; ^4^UCD-Centre for Food Safety, School of Public Health, Physiotherapy and Sports Science, University College Dublin Dublin, Ireland

**Keywords:** *P. zopfii*, bovine mastitis, bMECs, apoptosis, SEM, TEM

## Abstract

*Prototheca zopfii* infections are becoming global concerns in humans and animals. Bovine protothecal mastitis is characterized by deteriorating milk quality and quantity, thus imparting huge economic losses to dairy industry. Previous published studies mostly focused on the prevalence and characterization of *P. zopfii* from mastitis. However, the ultrastructural pathomorphological changes associated with apoptosis in bovine mammary epithelial cells (bMECs) are not studied yet. Therefore, in this study we aimed to evaluate the *in vitro* comparative apoptotic potential of *P. zopfii* genotype-I and -II on bMECs using flow cytometry, scanning electron microscopy (SEM), and transmission electron microscopy (TEM). The results showed fast growth rate and higher adhesion capability of genotype-II in bMECs as compared with genotype-I. The viability of bMECs infected with *P. zopfii* genotype-II was significantly decreased after 12 h (*p* < 0.05) and 24 h (*p* < 0.01) in comparison with control cells. Contrary, genotype-I couldn't show any significant effects on cell viability. Moreover, after infection of bMECs with genotype-II, the apoptosis increased significantly at 12 h (*p* < 0.05) and 24 h (*p* < 0.01) as compared with control group. Genotype-I couldn't display any significant effects on cell apoptosis. The host specificity of *P. zopfii* was also tested in mouse osteoblast cells, and the results suggest that genotype-I and -II could not cause any significant apoptosis in these cell lines. SEM interpreted the pathomorphological alterations in bMECs after infection. Adhesion of *P. zopfii* with cells and further disruption of cytomembrane validated the apoptosis caused by genotype-II under SEM. While genotype-1 couldn't cause any significant apoptosis in bMECs. Furthermore, genotype-II induced apoptotic manifested specific ultrastructure features, like cytoplasmic cavitation, swollen mitochondria, pyknosis, cytomembrane disruption, and appearance of apoptotic bodies under TEM. The findings of the current study revealed that genotype-II has the capability to invade and survive within the bMECs, thus imparting significant damages to the mammary cells which result in apoptosis. This study represents the first insights into the pathomorphological and ultrastructure features of apoptosis in bMECs induced by *P. zopfii* genotype-II.

## Introduction

Bovine mastitis is an inflammatory condition of mammary gland which is characterized by pathological, physiological, and bacteriological changes in the udder that affects the milk quality and quantity (Sharma et al., 2011). Different pathogens are involved in bovine mastitis which invades the udder through the teat canal in milking animals. These pathogens proliferate into the mammary gland and produce harmful effects which consequences in inflammation of udder (Seegers et al., 2003; Halasa et al., 2007). Protothecosis, mostly caused by *Prototheca zopfii*, clinically appears in the form of mastitis in dairy cattle (Ahrholdt et al., 2012). Bovine mammary protothecosis leads to subclinical and clinical bovine mastitis. In acute clinical form, protothecal mastitis is generally characterized by elevated body temperature (up to 40°C), pain, hot edema of the udder, loss of appetite, and reluctance to move. The chronic form of protothecal mastitis is accompanied by slight pain, hard tissue consistency with pasty edema in the udder, as well as pronounced decrease in milk production and there is elevated somatic cell count, especially macrophages, which may even lead to culling of cow and ultimately result in high economic losses (Wawron et al., 2013). The somatic cells are mainly cells of the immune system which mostly include leukocytes (75%), that is neutrophils, macrophages, lymphocytes, polymorphonuclear cells, and the disrupted mammary epithelial cells (25%) (Pillai et al., 2001). The leukocytes are part of natural defense mechanism to fight the infection and to help in the repair of damaged tissues. The epithelial cells of mammary gland are normally sloughed off and renewed, but in infection the number considerably increase. Somatic cells count is an important indicator of intramammary infection (IMI) as well as the quality of milk for human consumption (Schukken et al., 2003).

*P. zopfii*, a fungus like alga, is divided into two genotypes (type-I and -II) according to biochemical, serological, and genetic assays (Roesler et al., 2006). Additionally, *P. zopfii* genotype-II, *P. wickerhamii*, and *P. blaschkeae* have been mainly associated with bovine mastitis (Marques et al., 2008; Capra et al., 2014); while, *P. wickerhamii* and *P. cutis* are mostly related to human diseases (Lass-Florl and Mayr, 2007; Satoh et al., 2010). Protothecosis is also important from public health point of view, as *P. zopfii* is usually associated with bovine mastitis which can be transferred to human being through consumption of contaminated milk (Bozzo et al., 2014). Cutaneous or disseminated infection and olecranon bursitis are main form of protothecosis in human being (Lass-Florl and Mayr, 2007).

Bovine udder tissue is the main target site of protothecal infection and ascending infection can lead to long-term involvement. Chronic mastitis caused by *P. zopfii* is dominated by macrophages in bovine mammary gland (Cheville et al., 1984). Their study reported that mammary gland infected with *P. zopfii* showed different degenerative stages of algal cells contained by macrophages in the interstitial spaces, seclude between alveolar epithelial cells and lumen of alveoli under electron microscopy (Cheville et al., 1984). Recent protothecal bovine mastitis outbreaks have been reported from China (Gao et al., 2012; Chang et al., 2013; Shahid et al., 2016), Canada (Pieper et al., 2012), Iran (Zaini et al., 2012), Japan (Sobukawa et al., 2012), Mexico (Mayorga et al., 2012), Poland (Jagielski et al., 2011), and Romania (Bouari et al., 2011); hence, it led to considerable economic losses in dairy herds. The findings of these studies investigated that genotype-II is the causative agent of bovine mastitis. However, there is still a paucity of published literature about the ultrastructure features of apoptosis in bMECs after *P. zopfii* exposure.

Apoptosis in cells after infection or any injury is characterized by typical ultrastructural features, such as shrinkage of cell, chromatin condensation, pyknosis, nuclear fragmentation, and appearance of apoptotic bodies (Gajewska et al., 2005). The invasion and survival of *P. zopfii* type-II in bMECs and its role in apoptosis is not comprehensively studied yet. Previous published studies regarding protothecal mastitis caused by *P. zopfii* are mostly focused on prevalence, molecular characterization of *P. zopfii* (Jagielski et al., 2010; Gao et al., 2012; Chang et al., 2013; Bozzo et al., 2014; Shahid et al., 2016), and on the determination of some immunogenic proteins and virulence determinants of *P. zopfii* genotype-II (Irrgang et al., 2015). Therefore, the current study was designed with the objective to evaluate the ultrastructural features of apoptosis and to study the comparative apoptotic potentials of *P. zopfii* genotype-I and -II on bMECs. To the best of our knowledge, this is the first *in vitro* study unveiling the ultrastructural features of apoptosis in bMECs infected with *P. zopfii* genotype-I and -II.

## Materials and methods

### Isolation and identification of *P. zopfii*

*P. zopfii* genotype-I and -II were previously isolated from milk samples of dairy cows suffering from bovine mastitis and were stored in our laboratory at College of Veterinary Medicine, China Agricultural University, Beijing (Gao et al., 2012). The strains were revived on sabouraud dextrose agar (SDA; Difco™, Becton Dickison, Sparks MD, USA) at 37°C for 72 h and were used to assess their ability to invade and to evaluate their apoptotic potential in bovine mammary epithelial cells (bMECs). Prior to each experiment, fresh *P. zopfii* type-I and -II suspensions were used as described formerly (Shahid et al., 2016). *P. zopfii* was very carefully handled and all the experimental steps were carried out in biological safety cabinets, and we ensured the biosafety level 2 (BSL 2) precautions according to guidelines of Centers of Disease Control and Prevention (CDC).

### Cell morphology of *P. zopfii*

Colonies of the *P. zopfii* genotype-I and -II were resuspended in phosphate buffered saline with pH 7.4 (PBS) and evaluated using an optical microscope (CKX 41SF Olympus, Japan).

### Colonial morphology

*P. zopfii* genotype-I and II were cultivated on SDA to observe the morphological characteristics of colonies, the cultured plates were incubated at 37°C under aerobic condition for 5–7 days.

### Exponential growth curve

The exponential growth curve features of *P. zopfii* were analyzed in triplicate. The strains of genotype-I and -II were inoculated into 10 mL sabouraud dextrose broth (SDB; Difco™, Becton Dickison, Sparks MD, USA) in 15 mL centrifuge tube incubated at 37°C on a rotary shaker (150 rpm). Algal growth was determined by counting CFU per 4 h for first 3 days, after that CFU was counted per 24 for 7–8 days on SDA.

### Cell culture

Primary bMECs were isolated and characterized as described previously (Liu et al., 2014). Cells were cultivated in growth medium containing Dulbecco's modified Eagle's (DMEM)/F12 medium (HyClone, USA) supplemented with 10% Fetal Bovine Serum (FBS; Gibco, Grand island, NY, USA) and antimicrobial agents (100 U/mL penicillin, 100 mg/mL streptomycin and 1 mg/mL amphotericin B) in cell culture plates (Corning, Corning, NY, USA). Cells were incubated in 5% CO_2_ at 37°C, and cells from 2–8 passages were used for experiments.

### Infection experiments

For infection experiments, *P. zopfii* genotype-I and -II were grown on SDA for 48 h at 37°C, single colony was sub-cultivated into SDB for 72 h, after that *P. zopfii* were collected and suspended in DMEM/F12. Finally, the concentrations genotype-I and -II was adjusted to 5 × 10^5^ CFU/mL for all experiments.

### Adhesion capability and survival of *P. zopfii* genotypes

Adhesion capability and time course of the survival of *P. zopfii* genotypes-I and -II in bMECs was determined as described previously (Pereyra et al., 2016; Chen et al., 2017). The bMECs were infected with genotypes-I and –II at a 5:1 multiplicity of infection (MOI; ratio of *P. zopfii* to bMECs). To study the adhesion capability and survival of *P. zopfii* genotypes-I and -II in bMEC cells, samples were taken at 1, 4, 8, 12, and 24 h and cultured on SDA after infection.

### Cell viability assay in bMECs

MTT cell proliferation assay kit (Trevigen, Gaithersburg, MD, USA) was used to assess the cell viability according to Verma et al. (2010) with slight modification. The bMECs at a density of 1 × 10^5^ cells per well were cultured in 96-well plates. After being treated as mentioned above, the cells were washed three times with PBS (pH 7.2), and cells were incubated with 100 μL medium and 10 μL of the activated MTT solution at 37°C for 4 h, after that treated with 50 μL DMSO (Sigma-Aldrich), and the absorption was measured by microplate reader (SpectraMax 190, Molecular Devices Corporation, Sunnyvale, CA, USA) at 570 nm. Cell viability was determined as the percentages (%) of the control.

### Apoptosis in bMECs

The infected bMECs with *P. zopfii* genotype-I and -II were stained with FITC annexin V/PI apoptosis detection kit (Becton Dickinson, Franklin Lakes, NJ, USA) as described previously (Liu et al., 2014). Apoptosis rate was expressed using a FACSAria flow cytometry (Becton Dickinson, USA).

### Detection of Bax expression in infected bMECs using western blotting

To further validate the apoptosis in bMECs caused by *P. zopfii* genotypes, Bax protein expression was detected by western blot analysis. The bMECs were infected with *P. zopfii* genotype-I and -II for 0, 4, 12, and 24 h at 37°C with 5% CO_2_. Total protein was extracted from protothecal infected cells with protein extraction kit (KeyGEN, Nanjing, China). The protein quantification was carried out using bicinchoninic acid (BCA) protein assay kit (Beyotime, Haimen, China). Equivalent proteins from each sample were separated by sodium dodecyl sulfate polyacrylamide gel electrophoresis (SDS-PAGE) and transferred to a polyvinylidene difluoride membrane (PVDF; Millipore, MA, USA). Subsequently, the membranes were blocked in 5% bovine serum albumin (BSA; Gibco) and incubated with specific primary antibody for Bax (1:200, Santa, USA) and β-actin (1:1,000, Cell Signaling Technology, USA) overnight at 4°C followed by incubation with HRP-conjugated secondary antibody (1:5,000) for 1 h at room temperature. Finally, the bands were visualized using an enhanced chemiluminescence system (ECL, Beyotime, Haimen, China). The results were normalized to β-actin using Image J (National Institutes of Mental Health, Bethesda, MD, USA).

### Apoptosis in mouse osteoblast cells

To explore the host cells specificity of *P. zopfii*, we infected the mouse osteoblast cell line MC3T3-E1 with *P. zopfii* genotype-I and -II. The MC3T3-E1 was purchased from American Type Culture Collection (ATCC, Rockville, MD, USA). These cell lines were maintained in minimal essential medium (α-MEM medium; Gibco) in the presence of 10% (v/v) fetal bovine serum (FBS; Gibco) and antimicrobials (100 U/mL penicillin and 100 U/mL streptomycin), incubated at 37°C in the presence of 5% CO_2_. The infection protocol and flow cytometry assay was same as described earlier.

### Scanning electron microscopy (SEM) and transmission electron microscopy (TEM) of bMECs

The infected cells with *P. zopfii* genotype-I and -II were washed three times with phosphate buffer saline (PBS; pH 7.2) and the cells were fixed with 2.5% glutaraldehyde (Novon Scientific, China; pH 7.4) as described previously (Chen et al., 2017). Then cells were dehydrated by a graded ethanol from 30 to 100%, dried by critical-point drying method, then gold-coated with E-1010 Ion Sputter Coater (Hitachi, Japan). Finally, the changes in bMECs were observed by SEM (TESCAN 5136, Czech Republic). TEM was used for ultrastructure analysis of bMECs infected with *P. zopfii* genotype-I and -II. The bMECs were cultured and challenged as mentioned above and after washing with PBS cells were fixed with 2% glutaraldehyde-1% paraformaldehyde solution (Sinopharm Chemical Reagent Co., Ltd., China; pH 7.2) for 45 min at room temperature. After washing with PBS, the fixed cells were harvested with a rubber scraper (Fisher Scientific, Nepean, ON, Canada) in 1.5 mL microcentrifuge tubes. Further steps in the processing of the TEM samples were conducted at Electron Microscopy Unit of Beijing University of Agriculture, China. Briefly, the pellet was washed 5–7 times with phosphate buffer saline (pH 7.2), post-fixed in a 0.5% osmium tetroxide for 2 h, washed 5–7 times with PBS. After five times washing with PBS, the pellet was dehydrated with graded alcohol series (30, 50, 70, 80, 90, 95, and 100%). The pellet was embedded in 50/50 LR white embedding resin (Electron Microscopic Sciences, Hatfield, PA, USA) and pure ethanol solution for 1 h followed by a pure resin solution for overnight at 4°C and incubated for 1 h at 60°C for polymerization. Thin slices (100 nm) were cut by an Ultracutmicrotome (Leica EM, Germany) with a glass knife. Sections were positioned on copper grid, stained with 2% uranyl acetate and lead citrate and were viewed on a transmission electron microscopy (HITACHI H-7650, Japan) at 80 kV. The imaging was done using a 4 K Gatan CCD camera using the iTEM software.

### Statistical analysis

Statistical evaluation for triplicate experiments was carried out using student's *t*-test. Data are presented as the mean values ± standard deviation. *p* < 0.05 was considered as significant, while *p* < 0.01 was highly significant.

## Results

### Morphological characteristics of *P. zopfii*

**Figure 1:**
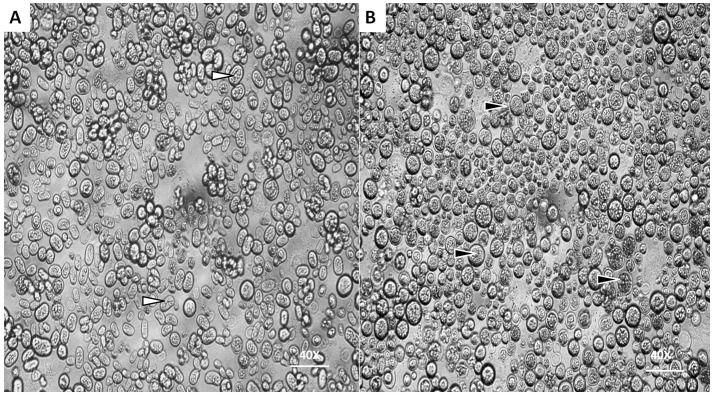
Morphological characteristics of *P. zopfii* under optical microscope. **(A,B)** Showing the different cells sizes of *P. zopfii* genotype-I (black arrow) and genotype-II (white arrow) with internally dividing cells with endospores (40x).

**Figure 2:**
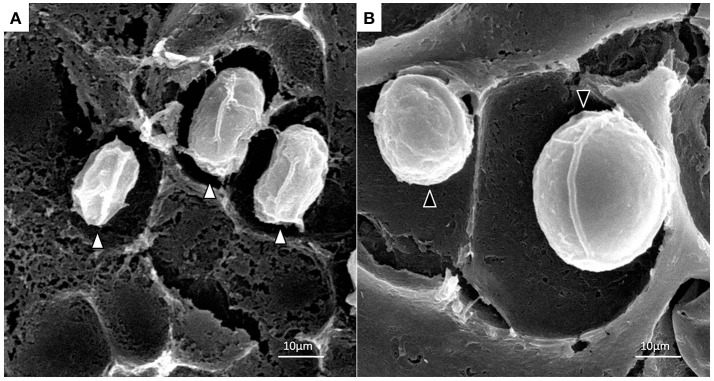
Scanning electron micrographs of *P. zopfii* genotype-II **(A)** and -I **(B)** cells. Indicating the difference in sporangial size and shape of genotype-I and -II.

Under optical microscope the cells of *P. zopfii* genotype-I were spherical in shape whereas that of genotype-II were oval, elliptical, and smaller in size than genotype-I. Figures 1A,B indicates the different phases of microbial growth, having numerous stem cells called sporangia, forming sporangiospores or endospores by internal division. Under the electron microscopy, there were different morphological feature of genotype-II and genotype-I sporangia. Genotype-II revealed typical features of sporangia with spherical to oval, containing sporangiospores as shown in Figure 2A; while type-I showed compact, round shaped and twice in size of type-II (Figure 2B).

### Colonial morphology

**Figure 3:**
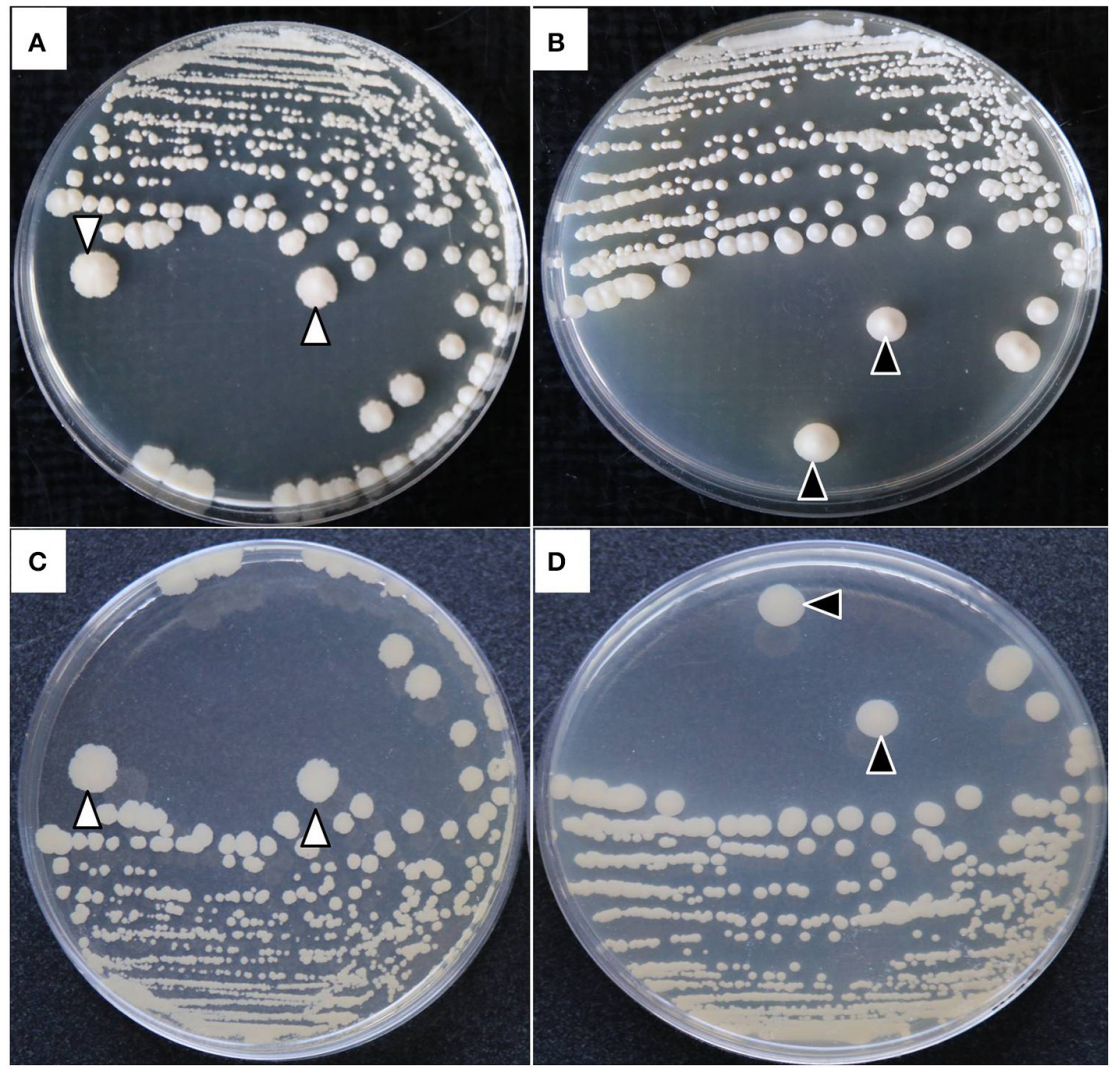
Colonial morphological characteristics of *P. zopfii* genotype-I and -II. **(A)** Type-II with central irregular protrusion like granular surface and serrated borders, **(B)** Type-I with smooth bulging surface at the center of colony. **(C,D)** Back view of the colonies.

The colonies of both *P. zopfii* showed typical morphological characteristics like in case of genotype-II grayish white in color, with a central protrusion with granular surface and serrated shape (Figures 3A,C), while genotype-I displayed the creamy white colonies with bulging smooth surface (Figures 3B,D).

### Exponential growth curve

**Figure 4:**
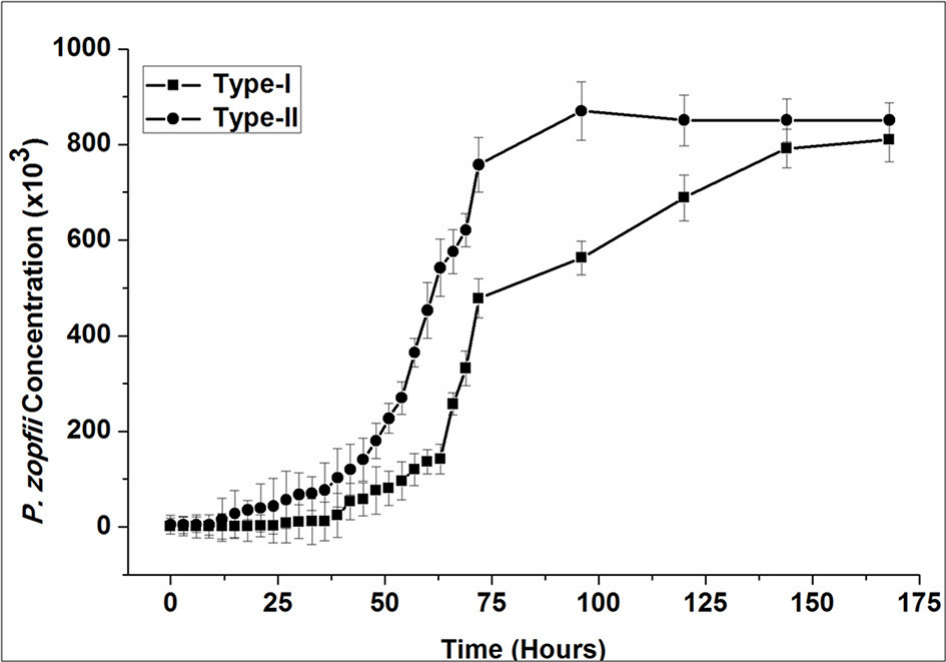
Exponential growth curve of *P. zopfii* genotype-I and -II. *P. zopfii* genotype-I and -II were initially grown on sabouraud dextrose agar (SDA; Difco™), sub-cultivated into sabouraud dextrose broth for 72 h. Exponential growth curve, as determined from colony forming unit, showed genotype-I and -II entered in logarithmic growth phase after 30 and 12 h and achieved the peak level at 144 and 96 h, respectively.

The exponential growth of *P. zopfii* is shown in Figure 4. According to growth curve of CFU, genotype-I and -II entered in logarithmic growth phase after 30 and 12 h and achieved the peak level at 144 and 96 h, respectively. This indicated that the genotype-II has faster growth kinetics than genotype-I.

### Survival of *P. zopfii* genotypes-I and -II in bMECs

**Figure 5:**
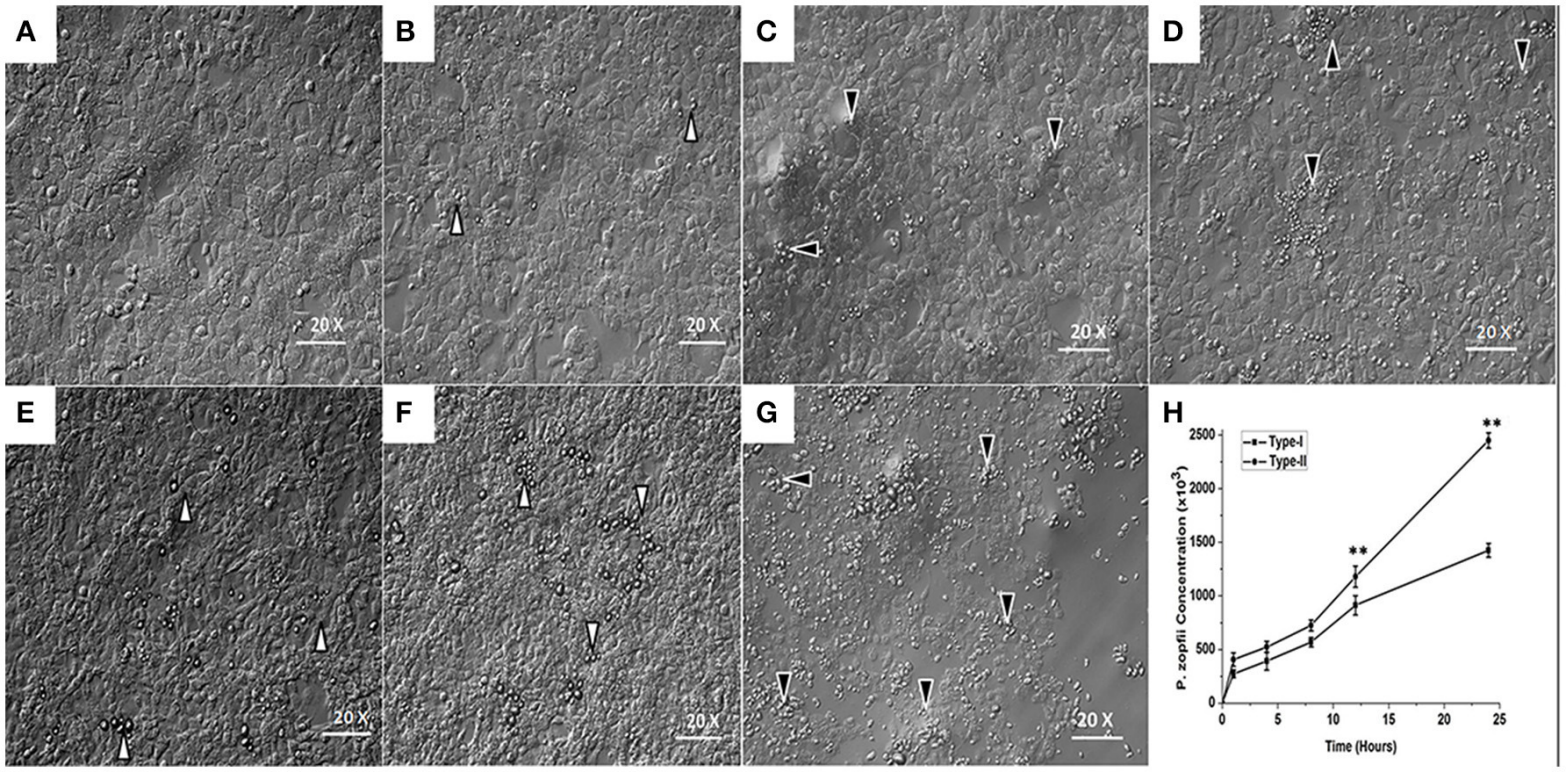
Adhesion of *P. zopfii* and its effect on bMECs morphology at different time of infection. **(A)** control, **(B,C)** 4 h, **(D,E)** 12 h, and **(F,G)** 24 h. Black arrowheads show the type-II, while white arrowheads show type-I cells adhesion. **(B**,**C,E**,**F)** There were no significant changes in bMECs, **(D)** Depicting slight cells morphological changes in type-II at 12 h, **(G)** Showing disruption of monolayer of bMECs in type-II at 24 h. **(H)** Time course of the survival and adhesive capability of *P. zopfii* in bMECs that increased significantly with passage of time. Data indicated as Mean ± *SD* of three independent experiments. ^**^p < 0.01.

Morphological changes in the bMECs monolayers following infection with genotype-I and -II are shown in Figures 5A–G. In case of genotype-I infection, the bMECs appeared similar to the control (uninfected cells); whereas type-II showed slight detachment from bMECs monolayer after 12 h (Figure 5F) and affected the integrity of monolayer cells after 24 h, as obvious from bMECs detachment in Figure 5G. The results depicted that the numbers of *P. zopfii* genotypes-I and -II increased steadily with passage of time as elaborated in Figure 5H. The genotype-II exhibited stronger adhesion capacity as compared to type-I.

### Cell viability

**Figure 6:**
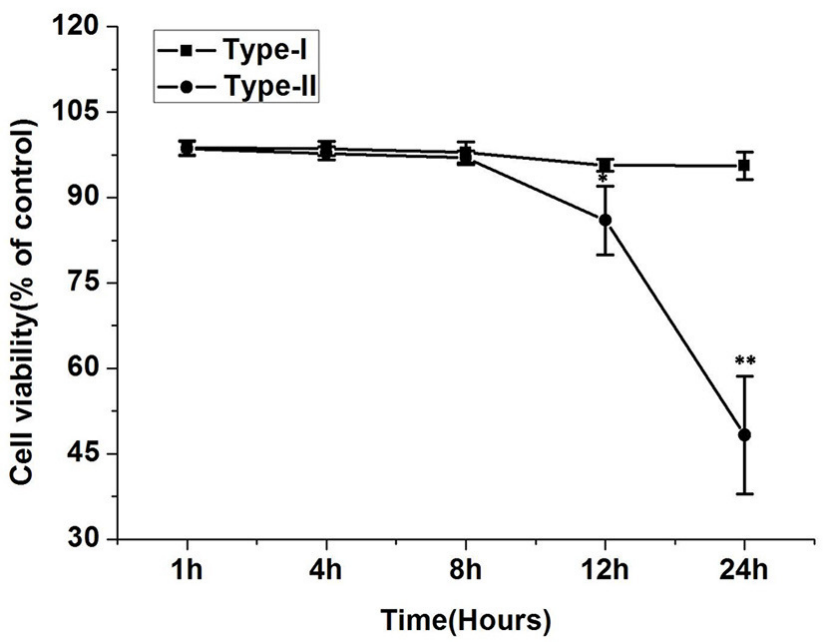
Effect of *P. zopfii* on the cells viability of bMECs by MTT assays. The viability of bMECs infected with *P. zopfii* genotype-II for 12 and 24 h was significantly decreased in comparison to uninfected group. Results are presented as Mean ± *SD*. ^*^p < 0.05, ^**^p < 0.01.

The results of the MTT assay revealed that viability of cells infected with *P. zopfii* genotype-II was significantly decreased after 12 and 24 h as compared with the uninfected cells (Figure 6), the cell viability values significantly declined from 85.3 ± 3.7% (*p* < 0.05) to 48.17 ± 10.38% (*p* < 0.01), respectively. In contrast, genotype-I didn't show significant effect on cell viability as compared with control.

### Apoptosis in bMECs

**Figure 7:**
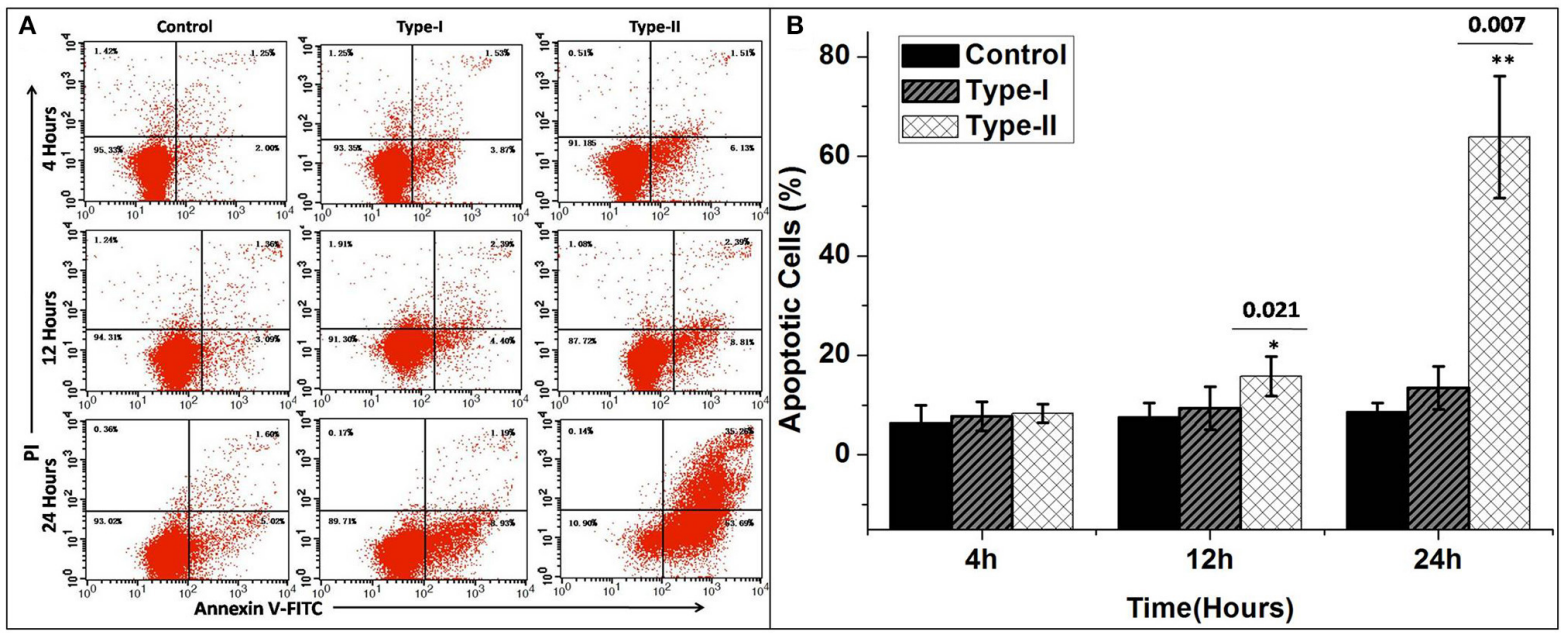
Apoptosis of bMECs induced by *P. zopfii*. Cells distributions were examined on basis of annexin V binding and propidium iodide (PI) dye uptake. The florescence was measured by flow cytometry. **(A)** Demonstrates two dimensional scatter plots of annexin V vs. PI. **(B)** Percentage of apoptotic cells. Control (uninfected group), type-I (5 × 10^5^ CFU/ml) infection, type-II (5 × 10^5^ CFU/ml) infection for 4, 12, and 24 h. Data of triplicate experiments are shown as Mean ± *SD*. ^*^p < 0.05, ^**^p < 0.01.

The results of flow cytometry assays showed significant changes in cell profile after exposure to *P. zopfii* genotype-II for 12 and 24 h. These results revealed that genotype-II significantly induced apoptosis in bMECs. After exposure, genotype-II showed high rise in apoptosis in comparison with control group as apoptotic rate was increased from 14.91 ± 5.54% (*p* < 0.05) to 63.83 ± 23.28% (*p* < 0.01) at 12 to 24 h, respectively. While, in case of genotype-I, there was non-significant effect on cells apoptosis (Figures 7A,B).

### Bax protein expression in bMECs after *P. zopfii* genotype -I and -II infection

**Figure 8:**
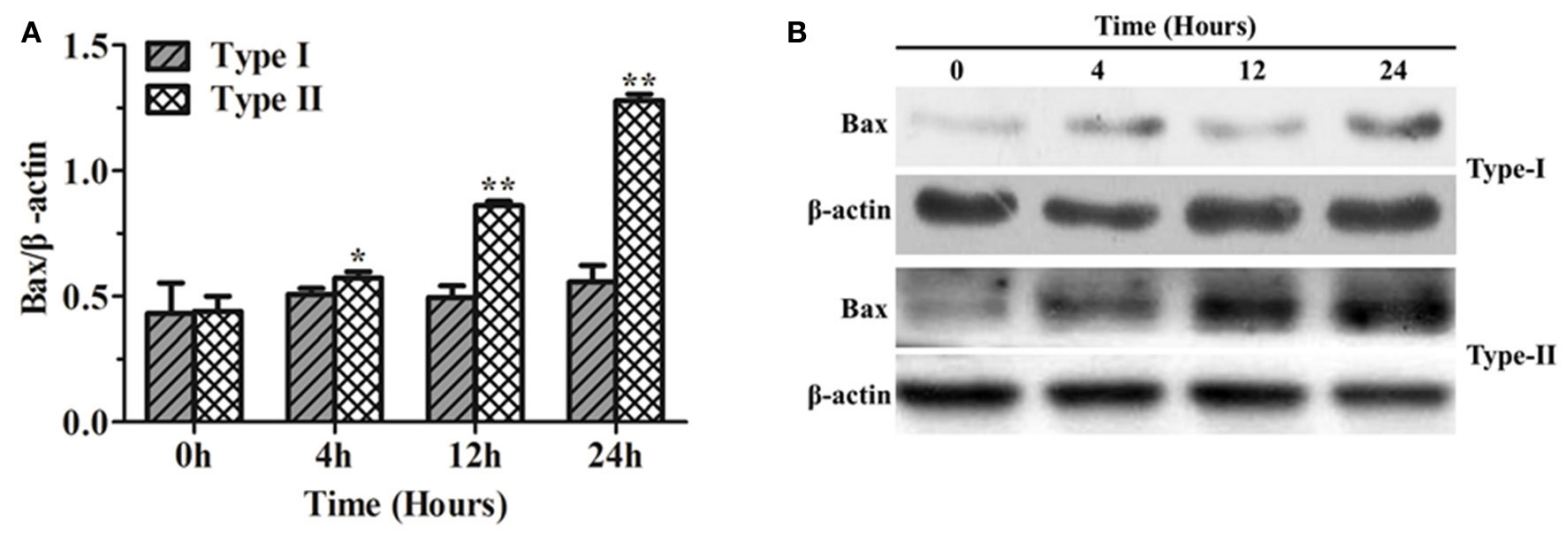
Bax protein expression in bMECs infected with *Prototheca zopfii* by western blot analysis. **(A)** *P. zopfii* genotype-II induces Bax protein expression. bMECs were treated with *P. zopfii* genotype-I and -II at 4, 12, and 24 h. **(B)** Bax protein levels were assessed by western blot analysis. Data are presented as mean ± *SD* of three separate experiments. ^*^p < 0.05, ^**^p < 0.01.

The results of Bax protein expression, using western blotting, in bMECs infected with *P. zopfii* genotype-I and -II are shown in Figure 8. Genotype-I showed non-significant effects on the expression Bax in bMECs as compared to control (i.e., at 0 h of infection). However, bMECs infected with genotype-II for 4, 12, and 24 h resulted in significant (*p* < 0.01) increase in Bax level (Figures 8A,B). This also validates the apoptotic effects of genotype-II on bMECs.

### Apoptosis in mouse osteoblast cells (MC3T3-E1)

**Figure 9:**
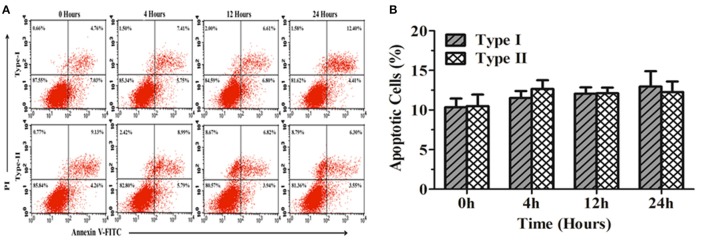
Apoptosis of mouse osteoblast cells (MC3T3-E1) induced by *P. zopfii*. **(A)** Represents two dimensional scatter plots of annexin V vs. PI. **(B)** Percentage of apoptotic cells. Control (0 h), genotype-I (5 × 10^5^ CFU/mL) infection, type-II (5 × 10^5^ CFU/ml) infection for 4, 12, and 24 h.

Figure 9 shows the apoptosis rate in MC3T3-E1 cells infected with *P. zopfii* genotype-I and -II for 0, 4, 12, and 24 h. The results elaborated non-significant difference in apoptosis rate in MC3T3-E1 cells at different time intervals (Figures 9A,B). This might suggest that *P. zopfii* genotype-I and -II didn't possess specificity toward these cell lines.

### Ultrastructural changes in bMECs infected with *P. zopfii*

**Figure 10:**
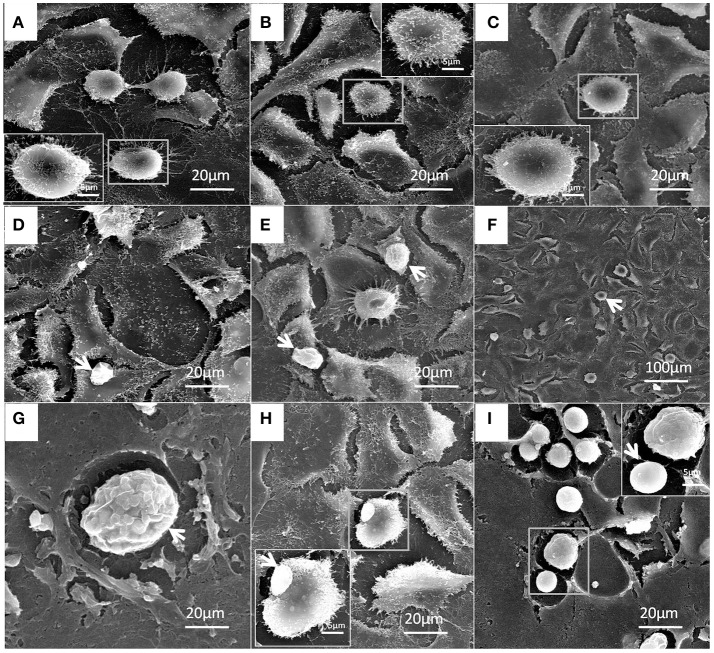
Patho-morphological alterations in bMECs after infection of *P. zopfii* genotype-I under SEM. **(A)** Control, **(B)** 4 h, and **(C)** 12 h showing no effect on cytomembrane of bMECs. **(D,E)** Cells adhesion of type-I with cells (white arrow) at 12 h. **(F–I)** Type-I infected epithelial cells at 24 h is depicting no morphological changes (white arrow). **(G)** Sporangia of type-I with many sporangiospores attached with the cells (white arrow). **(H)** Type-I attached with the cells (white arrow). **(I)** Different developmental stages of sporangia and sporangiospores (white arrow).

**Figure 11:**
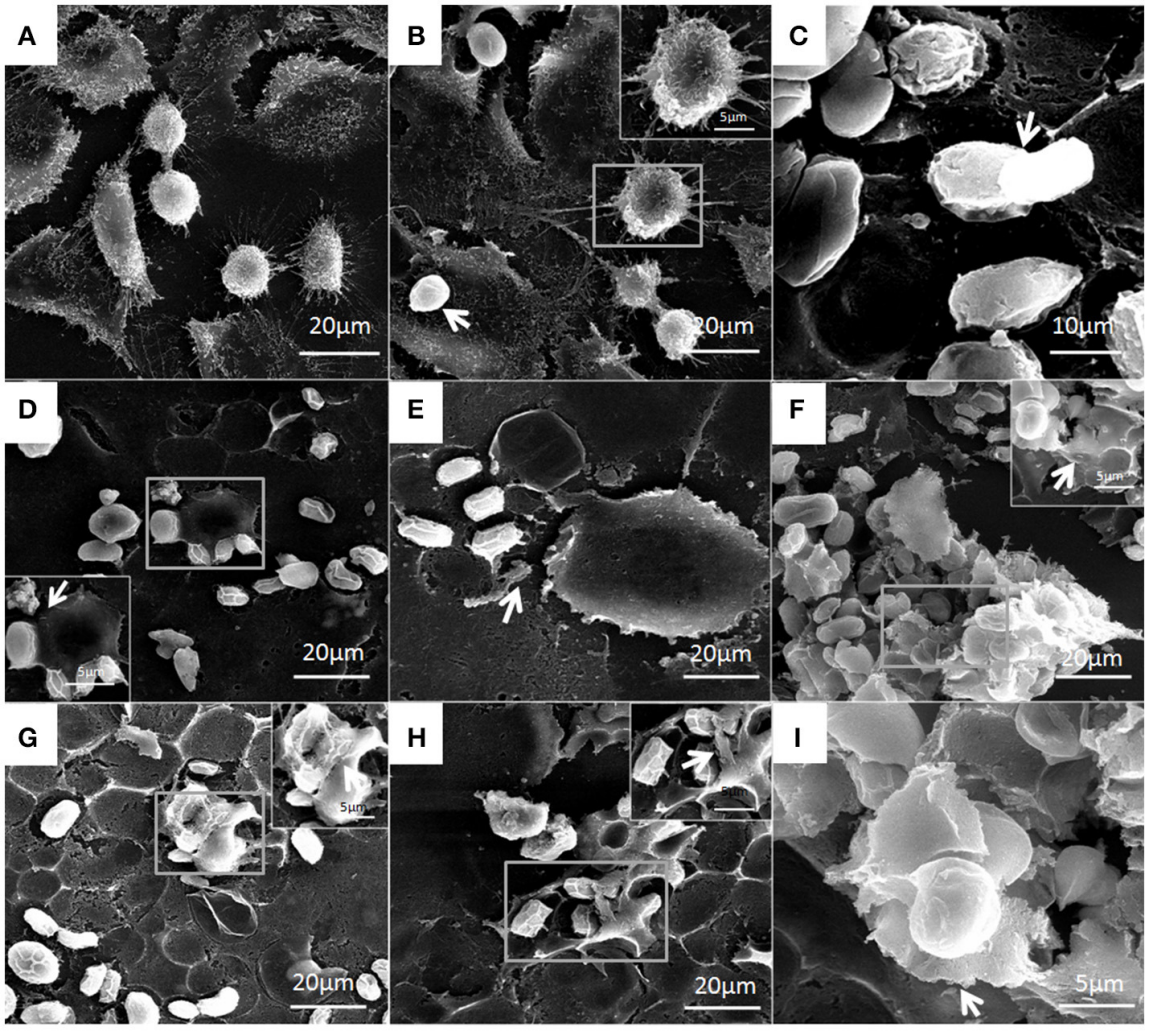
Structural features of bMECs after infection of *P. zopfii* genotype-II observed under SEM at 4, 12, and 24 h. **(A)** Control, **(B)** Shows no effect on cytomembrane but type-II shows the cells adhesion at 4 h (white arrow), and **(C)** Indicates the cells adhesion of sporangiospores and sporangia at 12 h (white arrow). **(D**,**E)** Depicts the start of apoptotic effect on cells at 12 h. **(F)** Represents the rapture of sporangia and shows sporopollenin (white arrow) after maturation and release of sporangiospores at 24 h. **(G–I)** Indicates the degree of cells disruption, disappearance of microvilli, and no more intact of cellular membrane (white arrow) at 24 h.

Under electron microscopy there were no morphological changes in uninfected bMECs (Figure 10A). In genotype-I infected group, there was also no prominent pathological alterations in the structure of cells; however the genotype-I have the adhesive capability with bMECs without damaging the cell integrity, this adhesion of genotype-I increased with the passage of time (Figures 10B–I). Whereas, genotype-II has showed adhesion without any structural alteration at 4 h as shown in Figure 11B. There were marked morphological changes in bMECs after exposure at 12–24 h. At 12 h, strong adhesion to bMECs was observed, which increased with passage of time, and disruption of cytomembrane initiated, this indicates the start of apoptosis (Figures 11C–F). At 24 h of exposure, the adhesion of genotype-II further increased (Figure 11G) and the cytomembrane was totally damaged (Figures 11H,I). In addition, the extent of cell disruption, the disappearance of microvilli and rupture of cellular membrane were prominent in the genotype-II exposure at 24 h (Figures 11G–I).

**Figure 12:**
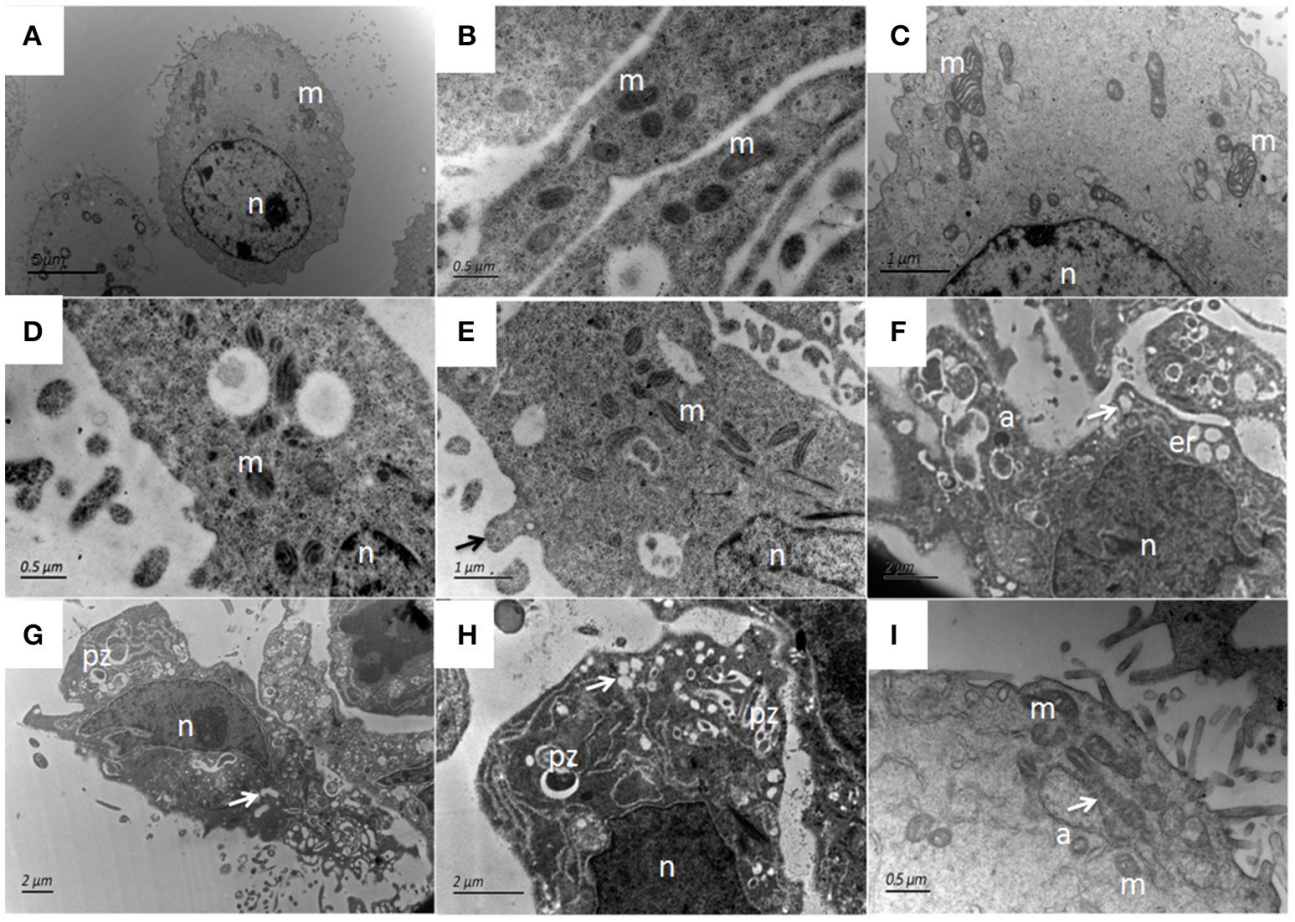
Ultrastructural pathological changes in bMECs after 4 h and 12 h infection of *P. zopfii* type-II under TEM. **(A–C)** Control bMECs. **(D)** Shows slight increase in size of mitochondria in bMECs at 4 h and **(E)** Depicts bulging (pseudopod) of bMECs toward *P. zopfii* type-II at 4 h (black arrow). **(F–H)** Vacuolization in cytoplasm at 12 h (white arrow) originated from the swelling of endoplasmic reticulum and mitochondria; with condense nucleus and appearance of apoptotic bodies. **(G,H)** Engulfment of algae by cells. **(I)** Showing higher magnification with swollen mitochondria (white arrow). pz, *P. zopfii;* n, nucleus; *m*, mitochondrion; *er*, endoplasmic reticulum; a, apoptotic bodies.

**Figure 13:**
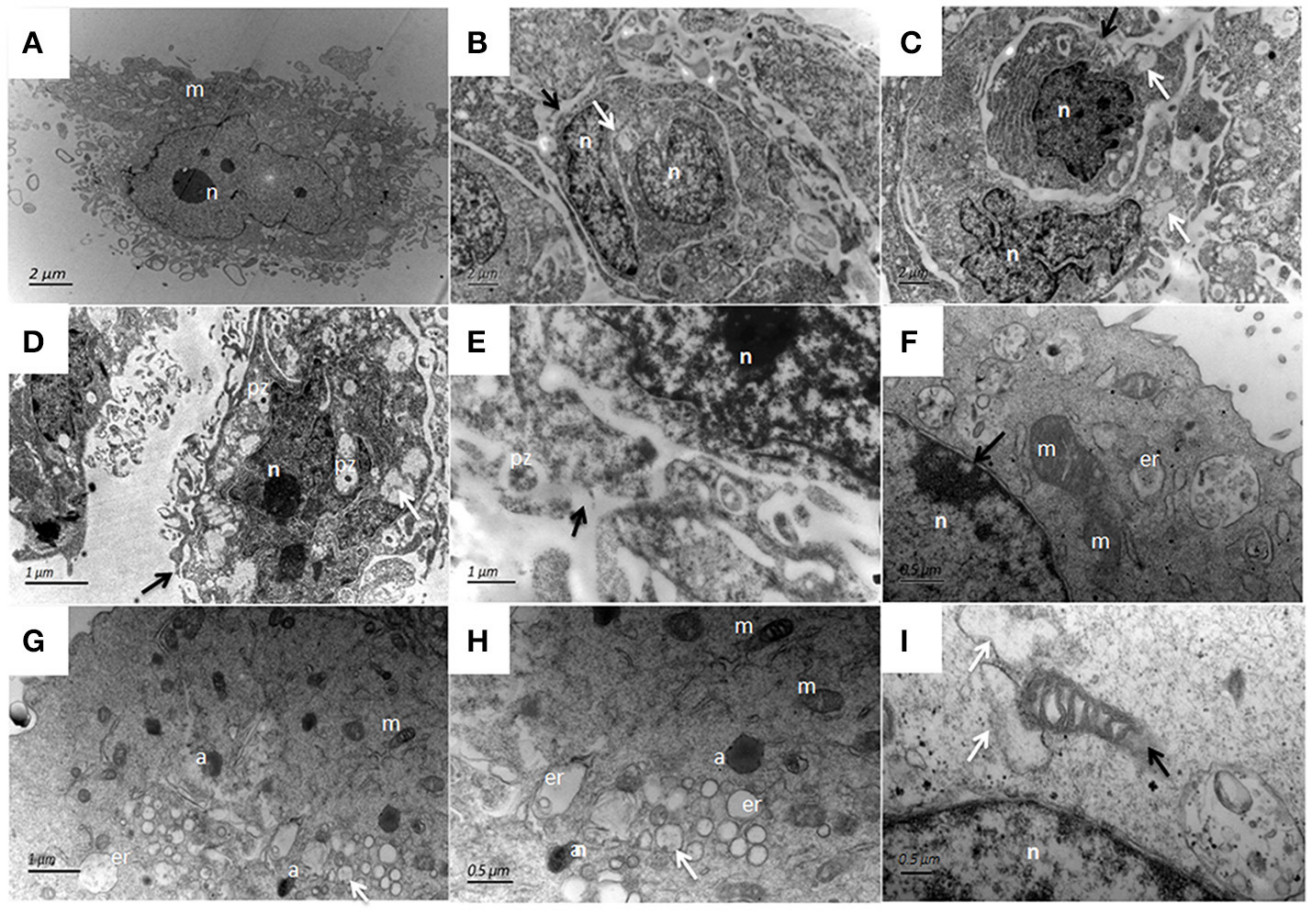
Patho-morphological changes of bMECs after 24 h infection with *P. zopfii* type-II under TEM. **(A)** Non-infected cells that show abundant mitochondria in cytoplasm, **(B,C)** Disruption of cytomembrane (black arrow), pyknosis of nucleus, swelling of endoplasmic reticulum as cytoplasmic vacuoles (white arrow). **(D,E)** Changes in cytoplasmic features, *P. zopfii* enclosed within the cytoplasmic vacuoles, the chromatin condensation, fragmentation, and cell membrane breakage (black arrow). **(F)** higher magnification indicating swollen mitochondria with crista degeneration and expansion of perinuclear space (black arrow). **(G)** Appearance of apoptotic bodies and swelling of endoplasmic reticulum as cytoplasmic vacuole. **(H)** higher magnification with apoptotic bodies. **(I)** Mitochondria disruption (black arrow) and cytoplasm replaced by phagocytic vacuoles (white arrow). pz, *P. zopfii;* n, nucleus; *m*, mitochondrion; *er*, endoplasmic reticulum; a, apoptotic bodies.

The ultrastructural features of bMECs as observed under TEM demonstrated various stages of *P. zopfii* genotype-II infection and its cytopathic and apoptotic effects in comparison with genotype-I. The normal bMECs maintained in DMEM with 10% FBS exhibited normal structure like intact cytoplasm and organelles (including intact mitochondria and endoplasmic reticulum structures) as in Figures 12A–C. After the adhesion of genotype-II at 4 h, slight increase in size of mitochondria was noted (Figure 12D), whereas, some epithelial cells extended around the adherent algae with the appearance of pseudopod like structures (Figure 12E). At 12 h, there were vacuolization in cytoplasm that originated from the swelling of endoplasmic reticulum as shown in Figure 12F and some of the mitochondria were swollen (Figure 12I). The genotype-II sporangiospores enclosed within the endocytic vacuoles were also seen in cells (Figures 12G–H). At 24 h post-infection, as elaborated in Figures 13B–I, all ultrastructural characteristics of apoptosis were confronted in the infected bMECs, such as cytoplasmic cavitation (Figures 13C,D,G), pyknosis (Figure 13D), cytomembrane disruption (Figures 13D,E), and appearance of apoptotic bodies (Figures 13G,H) as compared to control group (Figure 13A). The monolayer of bMECs was damaged and the released algae were seen near the remnant of disrupted epithelial cells as shown in Figure 13E at 24 h. The swollen mitochondrial structures and mitochondrial disruption were also observed (Figures 13F,H,I), and the cytoplasm was replaced by phagocytic vacuoles (Figure 13I) at 24 h after *P. zopfii* genotype-II infection. The average increase in mitochondrial size was 1.11, 1.69, and 2.63 μm at 4, 12, and 24 h post infection, respectively, as compared to the mitochondrial size of 0.98 μm of the control bMECs. Whereas, some epithelial cells having large vacuoles without any algae were observed, these vacuoles were the swelling of the endoplasmic reticulum (Figures 13F–H).

**Figure 14:**
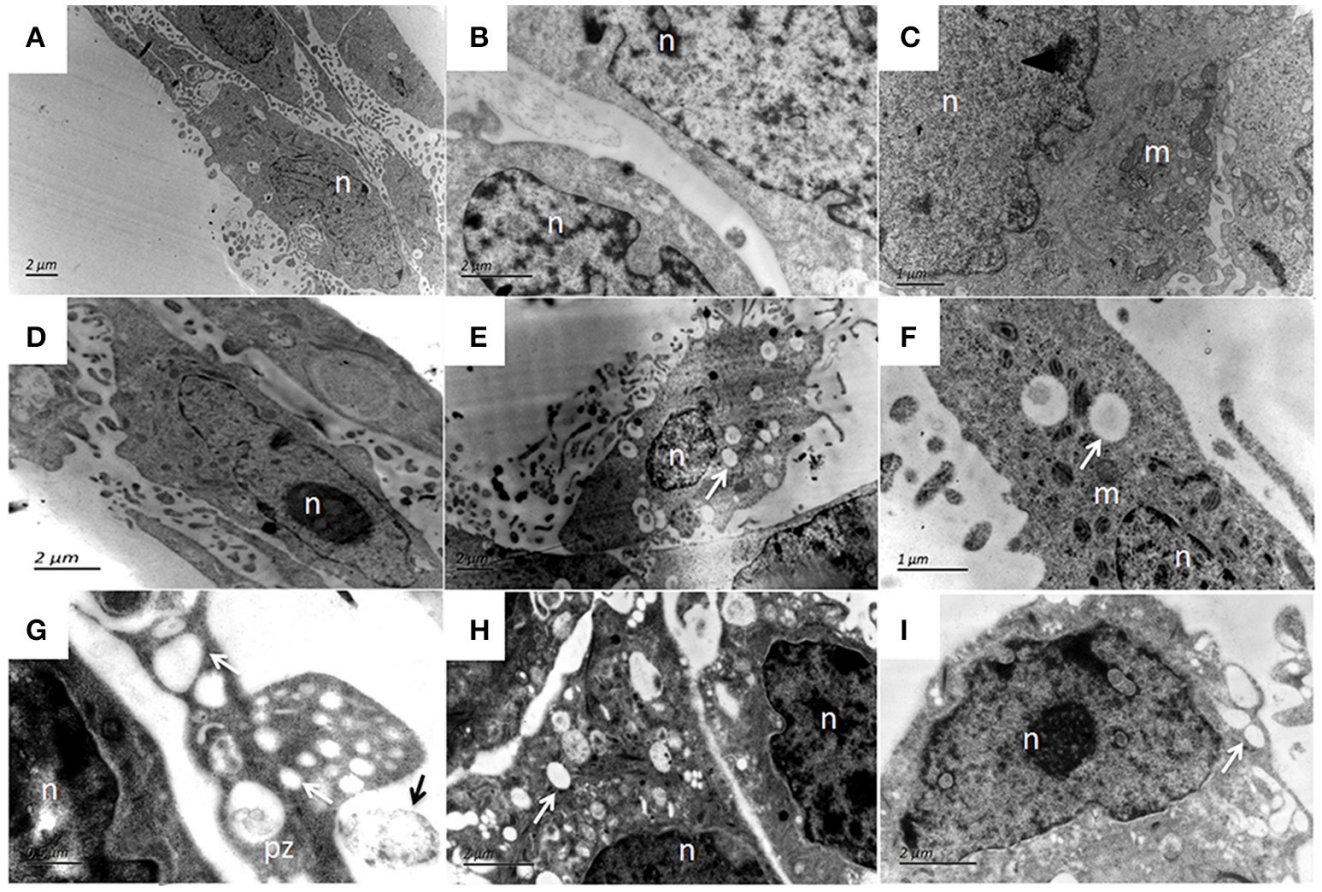
Ultrastructural features of bMECs after *P. zopfii* genotype-I infection at different time interval under TEM. **(A,B)** Uninfected cells with mitochondria, intact cytoplasmic, and nuclear membrane. **(C)** Cells at 4 h and **(D)** Cells at 12 h indicating no morphological changes in bMECs. **(E–I)** TEM of *P. zopfii* type-I infected cells at 24 h showing cytoplasm cavitation (white arrow), **(G,H)** Sporangiospores enclosed within the vacuoles, pseudopod like bulging surrounding sporangiospores and **(G)** Adherence of algae (black arrow) to epithelial cells surface. pz, *P. zopfii;* n, nucleus; *m*, mitochondrion; *er*, endoplasmic reticulum.

In *P. zopfii* genotype-I exposure, there was no marked ultrastructural changes in the bMECs at 4 h (Figure 14C) and 12 h (Figure 14D), similar like control cells (Figures 14A,B). While at 24 h post-infection the cytoplasmic membrane remained intact (Figures 14E,F,I), with slight vacuolization with enclosed algae (Figures 14G,H).

## Discussion

Chronic bovine mastitis caused by *P. zopfii* is increasingly reported in dairy herds and is liable for huge economic losses in dairy herds (Bozzo et al., 2014). Protothecosis is also important from public health point of view, as *P. zopfii* could be transmitted to humans through contaminated milk and cause intestinal infection like enteritis (Melville et al., 1999). The present study reported the ultrastructural features of apoptosis and to study the cytotoxic and apoptotic potentials of *P. zopfii* genotype-I and -II on bMECs. It also elucidated the morphological changes in bMECs after *P. zopfii* genotype-II *in vitro* infection under scanning electron microscope, which explored the relationship of the genotypes with the etiology of protothecal bovine mastitis and demonstrated that genotype-II caused severe apoptosis in bMECs in time dependent manner. As SEM depicted the adhesion of *P. zopfii* to the host cells which is the most important step in the infection process followed by colonization of the pathogen within the host cells. This is in agreement with previous studies (Melchior et al., 2006; Akers et al., 2015), which explored the adhesion and biofilm formation of *P. zopfii* associated with mastitis. Here, we comprehensively studied, for very first time, the pathomorphological and ultrastructure features of apoptosis in bMECs infected with *P. zopfii* genotype-II. Although, chronic protothecal mastitis causes increase in the somatic cell counts which is predominated by macrophages (Cheville et al., 1984); however, bMECs are of special importance in the mammary gland and its defense mechanism. These bMECs are at front line defense, play an important role in immune response of the mammary gland and can quickly respond against the invading pathogens (Gilbert et al., 2013). Thus, keeping the importance of bMECs in the udder health and the increasing prevalence of protothecal mastitis, this study was designed. Kwiecinski also recommended further studies for detail mechanism of apoptotic effects and control of protothecosis (Kwiecinski, 2015).

The results of the present study confirmed that *P. zopfii* genotype-II have higher apoptotic potential in bMECs. It is in agreement with the reports of Moller et al. in Germany (Moller et al., 2007), Aouay et al. in Belgium (Aouay et al., 2008), Osumi et al. in Japan (Osumi et al., 2008), Jagielski et al. in Poland (Jagielski et al., 2010), and Shahid et al. in China (Shahid et al., 2016). We determined Bax protein expression, by western blotting, to further validate the apoptosis in bMECs infected with *P. zopfii* genotypes. Our results showed that *P. zopfii* genotype-II for 4, 12, and 24 h resulted in a significant (*p* < 0.01) increase of Bax level. These finding supported our previous report that *P. zopfii* genotype-II induced apoptosis through reactive oxygen species (ROS) generation (Shahid et al., 2017). Increasing ROS level might activate the mitochondrial Bax that lead to apoptosis in bMECs after *P. zopfii* genotype-II infection. Bax is a proapoptotic factor of the Bcl-2 family proteins, and it plays an important role in the mitochondrial apoptosis pathway (Li et al., 2013). In normal living cells, Bax exists predominantly in the cytosol, and during apoptosis it migrates to the membrane of mitochondria (Yu et al., 2008). Our findings support these results that increase level of Bax expression in *P. zopfii* genotype-II infection induced apoptosis. In addition, the host specificity of genotype-II was also tested in mouse osteoblast cell lines (MC3T3-E1) and the results revealed that both genotype-I and -II could not cause any significant apoptosis in these cells. This might suggest that genotype-II does not have specificity toward the MC3T3-E1 cell lines.

In the current study, we examined ultrastructural variations in bMECs after *P. zopfii* genotype-I and -II exposure. *P. zopfii* genotype-II may proliferate in the bMECs and this proliferative stage may survive for some time following phagocytosis that was demonstrated by the observation of intracellular sporangia after 12 h of infection. Whereas, in genotype-I only few sporangia were observed inside the epithelial cells after 24 h of infection. The ultrastructure variations noted in the current study are compatible with the morphological features of apoptosis and para-apoptosis (Asher et al., 1995). Asher et al. (1995) were the first who described the paraptosis, which is characterized by cytoplasmic vacuolization due to swollen mitochondria and endoplasmic reticulum. Moreover, Sperandio et al. explained the form of para-apoptosis, called paraptosis, with similar ultrastructure features by cellular characteristics and response to inhibitors of apoptosis (Sperandio et al., 2000).

In the natural infection of protothecosis, it was observed that macrophages are sites for proliferation of *P. zopfii* and survives for some time after phagocytosis as observation of intact intracellular sporangia (Jensen et al., 1998). In an experimental infection of *Prototheca* in the BALB/c mice demonstrated that cells with whole organism have spindle shaped and/or oval nuclei with abundant dilated rough endoplasmic reticulum. Cells near the completion of pathogen digestion, presented irregular and/or independent nuclei with often one or two distinct nucleoli and also the cytoplasm was often replaced by phagocytic vacuoles (Horiuchi and Masuzawa, 1995). Electron microscopy of mammary gland from cows infected with *P. zopfii* elaborated that the macrophages with algae were distinctly enlarged due to primarily from reduplication of the Golgi complexes and its associated vesicles. This study also described that intracellular *P. zopfii* was degenerated and comprised of intact cell wall profiles that contained membrane fragment without nuclei and cytoplasmic organelles (Cheville et al., 1984). This is in line with the current findings that *P. zopfii* genotype-II survives within the bMECs and cause pathogenic effects, such as cytoplasmic cavitation, swollen mitochondrial structures, pyknosis, cytomembrane disruption, and appearance of apoptotic bodies.

In our study, the results of cell viability, apoptosis, scanning electron microscopy, and transmission electron microscopy depicted that both genotype-I and -II were persistent in bMECs, but the *in vitro* pathogenic effect of genotype-II were more profound as previously reported *in vivo* infection (Chang et al., 2013).

It is concluded that *P. zopfii* genotype-II have the capability to invade and survive within the bMECs, causing pathomorphological alterations associated with apoptosis in infected bMECs. Our findings support the previous observations that *P. zopfii* genotype-II is the causative agents of bovine mastitis (Jensen et al., 1998; Morandi et al., 2016). To date, there is no treatment to profoundly eliminate chronic mastitis in dairy cow herds; therefore, further investigative study should be carried out to understand the pathogenesis of chronic mastitis caused by *P. zopfii* genotype-II.

## Author contributions

Designed the research: BH and MS; performed the experiments: MS, JW, WC, TA, XG, DH, and RY; analyzed the molecular data: MS and together with JG; Wrote the paper: MS with collaboration of JW and RY; and revised and corrected the manuscript: SF. All authors have read and approved the final manuscript.

### Conflict of interest statement

The authors declare that the research was conducted in the absence of any commercial or financial relationships that could be construed as a potential conflict of interest.
